# Hexagonal BN‐Assisted Epitaxy of Strain Released GaN Films for True Green Light‐Emitting Diodes

**DOI:** 10.1002/advs.202000917

**Published:** 2020-09-27

**Authors:** Fang Liu, Ye Yu, Yuantao Zhang, Xin Rong, Tao Wang, Xiantong Zheng, Bowen Sheng, Liuyun Yang, Jiaqi Wei, Xuepeng Wang, Xianbin Li, Xuelin Yang, Fujun Xu, Zhixin Qin, Zhaohui Zhang, Bo Shen, Xinqiang Wang

**Affiliations:** ^1^ State Key Laboratory for Mesoscopic Physics and Frontiers Science Center for Nano‐optoelectronics School of Physics Peking University Beijing 100871 P. R. China; ^2^ State Key Laboratory of Integrated Optoelectronics College of Electronic Science and Engineering Jilin University Changchun 130012 P. R. China; ^3^ Electron Microscopy Laboratory School of Physics Peking University Beijing 100871 P. R. China; ^4^ Collaborative Innovation Center of Quantum Matter Beijing 100871 P. R. China

**Keywords:** 2D materials, III‐nitrides, growth mechanisms, hexagonal boron nitride, light‐emitting diodes

## Abstract

Epitaxial growth of III‐nitrides on 2D materials enables the realization of flexible optoelectronic devices for next‐generation wearable applications. Unfortunately, it is difficult to obtain high‐quality III‐nitride epilayers on 2D materials such as hexagonal BN (h‐BN) due to different atom hybridizations. Here, the epitaxy of single‐crystalline GaN films on the chemically activated h‐BN/Al_2_O_3_ substrates is reported, paying attention to interface atomic configuration. It is found that chemical‐activated h‐BN provides B—O—N and N—O bonds, where the latter ones act as effective artificial dangling bonds for following GaN nucleation, leading to Ga‐polar GaN films with a flat surface. The h‐BN is also found to be effective in modifying the compressive strain in GaN film and thus improves indium incorporation during the growth of InGaN quantum wells, resulting in the achievement of pure green light‐emitting diodes. This work provides an effective way for III‐nitrides epitaxy on h‐BN and a possible route to overcome the epitaxial bottleneck of high indium content III‐nitride light‐emitting devices.

## Graphical abstract

Epitaxy of single‐crystalline GaN films is performed on hexagonal BN (h‐BN)/sapphire substrates. This success is achieved through chemical activation on h‐BN, which generates N—O bonds, and thus provides nucleation sites for Ga‐polar GaN. Subsequently, pure green InGaN‐based light‐emitting diodes are fabricated with a considerable increase of indium incorporation, manifesting a great potential in long wavelength light‐emitting diodes.

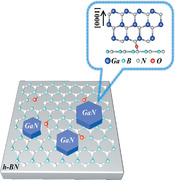

Recently, epitaxial growth of sp^3^‐hybridized III‐nitrides on sp^2^‐hybridized 2D materials has attracted considerable attentions.^[^ ^1^, ^2^, ^3^, ^4^, ^5^ ^]^ Those 2D materials are believed to minimize lattice mismatch between the epilayer and heterosubstrates due to the weakly bonded 2D interlayers.^[^ ^6^, ^7^, ^8^ ^]^ It is worth noting that the mismatch problem is one of the main challenges in realizing high‐efficiency long wavelength light‐emitting diodes (LEDs), such as green LEDs.^[^ ^9^, ^10^ ^]^ On the one hand, the lattice mismatch causes larger residual stress in the multiple quantum wells (MQWs) region, which limits the incorporation of indium (In) in the MQWs.^[^ ^11^, ^12^ ^]^ On the other hand, the larger residual stress introduces bigger polarization electric field in the MQWs region, resulting in a stronger quantum confinement Stark effect (QCSE), which significantly reduces the luminous efficiency of the green LED.^[^ ^13^ ^]^ Therefore, the epitaxy of nitrides on 2D materials is considered to be one of the potential methods for preparing high‐efficient green LEDs. Besides, epitaxy of III‐nitrides on 2D materials also shows its advantage for mechanically transferring epitaxial structures onto foreign substrates, thereby obtaining flexible III‐nitride based devices at a low cost and vertical devices as well.^[^ ^14^, ^15^ ^]^

Notably, as a family member of III‐nitrides, hexagonal BN (h‐BN) owes better growth compatibility than other 2D materials for the epitaxy of III‐nitride films and is thus believed to be the most suitable 2D material for III‐nitrides epitaxy.^[^ ^16^, ^17^ ^]^ However, the integration between h‐BN and conventional III‐nitride film still remains a challenge due to the difficulty in combining different atomic hybridization.^[^ ^18^, ^19^, ^20^, ^21^ ^]^ In fact, it is very difficult to form covalent bonds on 2D materials due to the absence of dangling bonds.^[^ ^22^, ^23^, ^24^, ^25^ ^]^ Therefore, nucleation of GaN on 2D materials is almost impossible except that the 2D materials are not perfect.^[^ ^26^, ^27^ ^]^ A couple of research groups tried to make dangling bonds or defects on 2D materials by using oxygen‐plasma pretreatment on the 2D materials to enhance the nucleation of GaN.^[^ ^28^, ^29^ ^]^ For example, Chung et al. reported the achievement of high‐quality GaN films on oxygen‐plasma‐treated graphene/Al_2_O_3_ substrates.^[^ ^1^ ^]^ Besides, Wu et al. reported the growth of AlGaN‐based deep‐ultraviolet LED structure on oxygen‐plasma‐treated h‐BN/Al_2_O_3_.^[^ ^30^ ^]^ Although several groups have reported the epitaxy of III‐nitrides on h‐BN, graphene, and so on, the interface bonding behaviors and/or the nucleation phenomena of III‐nitrides are seldom reported and are not well studied to the best of our knowledge.^[^ ^31^, ^32^, ^33^ ^]^

In this work, we study the epitaxy of GaN on 2‐in. h‐BN/Al_2_O_3_, and focus on the interface bonding and nucleation behavior. A chemical activation method is specially used to generate N—O bonds to facilitate the nucleation of GaN on the h‐BN surface and thus improve the crystal perfection for epilayers. It is found that the artificially added N—O bonds not only create more sites for following GaN nucleation but also modify the lattice polarity of GaN to be uniform Ga‐polarity one, leading to high‐quality material with flat surface. Based on those smooth GaN, we are then able to grow high‐quality InGaN quantum wells structure for green LEDs. That h‐BN layer is found to relax the compressive strain in GaN film and thus improve indium incorporation during the growth of InGaN quantum wells, leading to the emission wavelength redshift and achievement of pure green light‐emitting diodes.

GaN epitaxy was performed on 2‐in. h‐BN grown on (0001) Al_2_O_3_ substrate. The typical thickness for the h‐BN is about 3 nm. As shown in **Figure** **1**a, a typical Raman scattering spectrum of the 3‐nm‐thick h‐BN shows an intense scattering peak at 1364 cm^−1^ with a full width at half‐maximum (FWHM) as small as 10.7 cm^−1^, indicating a sp^2^‐hybridized BN with good crystal quality. The h‐BN shows wrinkle‐like morphology with a root mean square value of 0.9 nm in a scanned area of 5 × 5 µm^2^, as depicted in Figure 1b.

**Figure 1:**
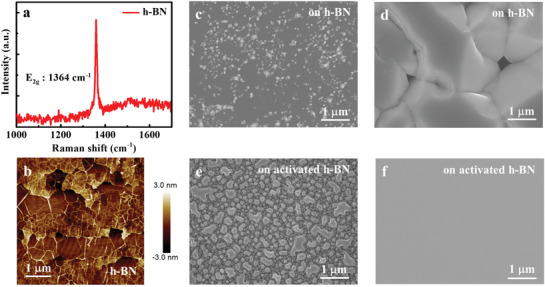
a) Raman scattering spectrum of 3‐nm‐thick annealed h‐BN on Al_2_O_3_. b) AFM image of 3‐nm‐thick annealed h‐BN on Al_2_O_3_. SEM images of c) 30‐nm‐thick LT‐GaN and d) subsequent 1.5‐µm‐thick HT‐GaN grown on untreated h‐BN/Al_2_O_3_ substrate. SEM images of e) 30‐nm‐thick LT‐GaN and f) subsequent 1.5‐µm‐thick HT‐GaN deposited on HCl‐activated h‐BN/Al_2_O_3_ substrate. These SEM results indicate that a drastically improved surface of HT‐GaN epilayer owes to the increasing of GaN nucleation density by HCl treatment of h‐BN.

Conventional two‐step growth was performed to deposit GaN films on h‐BN/Al_2_O_3_, which had achieved great success in the epitaxy of III‐nitrides on sp^3^‐bonded single crystal substrates such as Al_2_O_3_.^[^ ^34^, ^35^ ^]^ Unfortunately, with a growth interruption after low temperature (LT)‐GaN annealing, only limited number of GaN nucleation islands next to wrinkles has been observed, as depicted in Figure 1c. Afterward, those islands cannot coalesce well during conventional high temperature growth process and the high temperature (HT)‐GaN film shows a discontinuous surface, as depicted in Figure 1d. It reveals that direct growth of GaN on h‐BN/Al_2_O_3_ is difficult, because the absence of dangling bonds in 2D h‐BN indeed hinders GaN nucleation, especially, the more perfect the 2D, the worse the nucleation of GaN.

Here, we propose a chemical activation method to generate dangling bonds on the surface of h‐BN and thus to improve the nucleation ability of sp^3^‐hybrided GaN. To activate the surface, h‐BN was treated by hydrochloric acid (HCl) and deionized water, which is expected to introduce the hydroxide (OH) and thus the oxygen bonds so that the nucleation sites can be created. As depicted in Figure 1e, more GaN crystalline grains are observed on the activated h‐BN surface. This is further proved by the grain density statistics (see Figure S2, Supporting Information). After HCl treatment, the GaN grain density increases by about threefold from 24 to 69 µm^−2^, indicating a significant increase of nucleation sites, i.e., more surface dangling bonds are generated on h‐BN surface. This facilitates the coalescence of HT‐GaN and further leads to the formation of continuous and smooth films, as shown in Figure 1f.

To reveal the surface‐activated mechanism of h‐BN after HCl treatment, X‐ray photoemission spectroscopy (XPS) measurements were carried to identify the surface chemical states on h‐BN. **Figure** **2**a,b presents that the chemical state of B 1s remains B—N (190.8 eV) and the B—O related chemical state (191.4 eV) appears after the HCl treatment.^[^ ^36^, ^37^ ^]^ Simultaneously, the chemical state of N 1s remains B—N (396.5 eV) and the N—O related chemical state (400.4 eV) are detected after the HCl treatment, as described in Figure 2c,d.^[^ ^38^, ^39^ ^]^ Meanwhile, XPS spectra of activated h‐BN show that the intensities of B 1s and N 1s peaks obviously decrease, which are mostly attributed to the degradation of lattice structure (see Figure S3, Supporting Information). These results indicate that the newly formed surface defects and the original defects will provide some oxygen‐related artificial dangling bonds on the h‐BN surface during HCl treatment.

**Figure 2:**
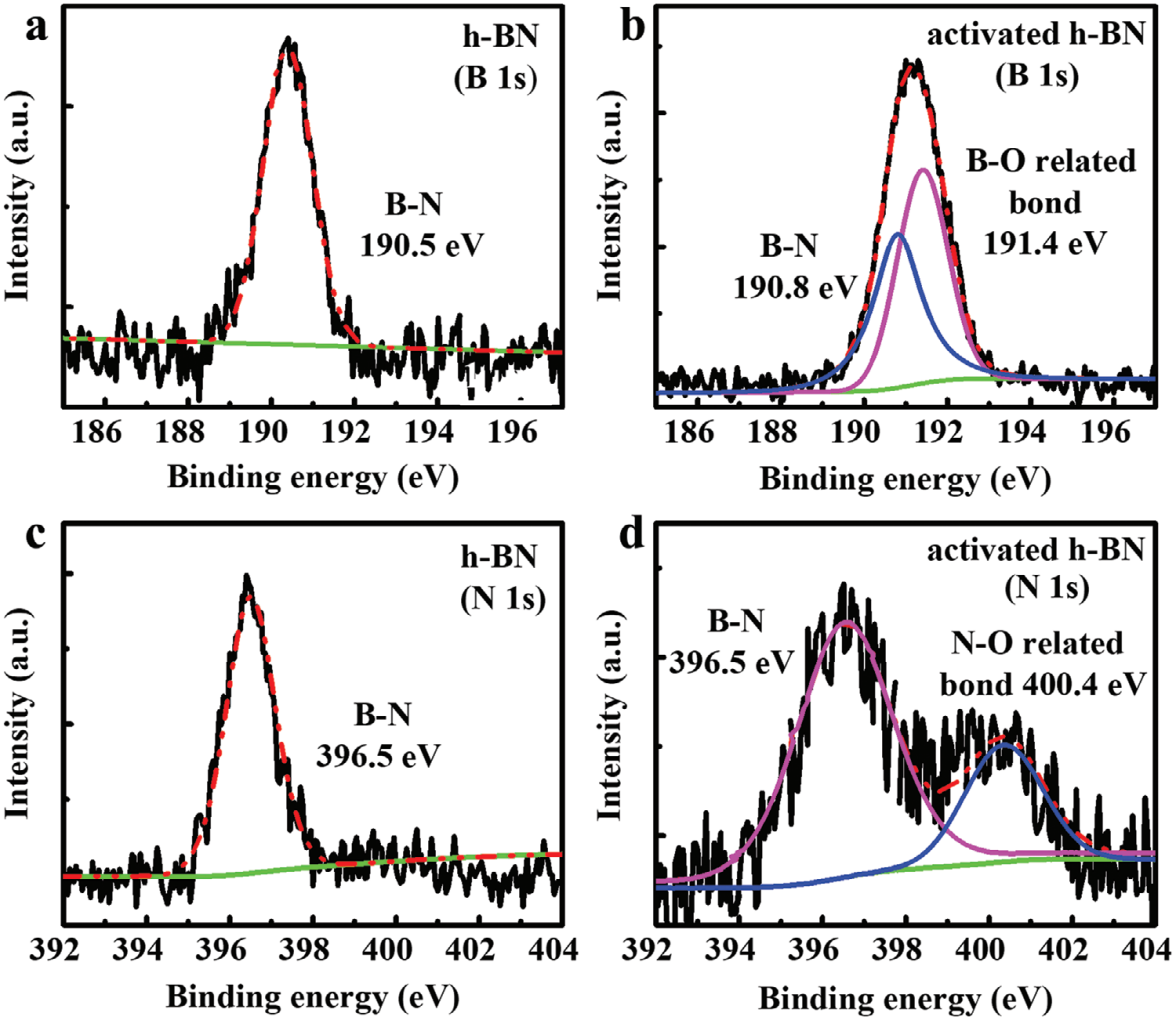
B 1s characteristic peak of h‐BN a) before and b) after HCl activation. The B 1s peak of activated h‐BN is consisted of B—N peak (190.8 eV) and B—O related peak (191.4 eV), showing the formation of O‐related defects bonding with B atoms. N 1s characteristic peak of h‐BN c) before and d) after HCl activation. The N 1s peak of activated h‐BN includes a contribution from peaks of B—N bond (396.5 eV) and N—O related bond (400.4 eV), which indicates some of newly formed O‐related defects bonding with N atoms. The thickness of h‐BN is about 3 nm.

Then, we performed first‐principles density functional theory (DFT) calculation to confirm the chemical adsorption process of O atoms on the h‐BN surface. As shown in **Figure** **3**a,b, four optimized chemical adsorption sites of single O atom on the surface of one monolayer h‐BN sheet are considered: floating in the center of the h‐BN honeycomb lattice (center), only bonding with B (B—O), only bonding with N (N—O), and bonding with B and N in the form of a bridge (B—O—N).^[^ ^32^, ^40^ ^]^ The binding energies between one O atom and h‐BN in these four structures are calculated to determine the most favorable chemisorbed O site on h‐BN, as shown in **Table** **1**. The calculation shows that the chemisorption of one O atom with B and N atoms forms a vertical B—O—N triangular ring, where it owes the largest binding energy (−2.101 eV). Here, the negative value means a binding. Besides, the configuration of N—O bond also provides a possible pathway because it not only displays a smaller lattice distortion of h‐BN sheet in this case but also holds the second largest binding energy (−1.566 eV). B−O bond shows a smaller binding energy (−1.142 eV), showing a worse chemical adsorption site for O atoms. The smallest binding energy (−0.316 eV) appears in the center position, where O atoms are normally unstable and tend to break away from the surface. Combined with the XPS results, it is believed that the B—O—N and N—O bonds are generated on the h‐BN surface. This is most likely due to that the sp^2^‐bonded h‐BN surface is not perfect, and there are some defective regions, such as vacancies and wrinkles. Due to the strong chemical activity of these defective regions, more surface defects can be induced during the treatment of HCl solution.^[^ ^41^, ^42^, ^43^ ^]^ At the same time, these surface defects can adsorb hydroxide ions in the HCl solution, forming two kinds of oxygen‐related defects, B—O and B—O—N bonds.^[^ ^44^, ^45^, ^46^ ^]^ Unfortunately, although the B—O—N bonds are the most stable according to the first‐principles DFT calculation, they are not easy to work as nucleation sites for GaN because B, O, and N atoms in the B—O—N bond are all in a saturated state. On the other hand, the O atoms in the N—O bonds are in an unsaturated state, and they are actually the most possible nucleation sites for GaN. Then, as shown in **Table** **2**, where the binding energies are calculated by the first‐principles DFT for the binding of Ga or N atoms to the N—O bonds and replacement of O atoms in the N—O bond by Ga or N atoms, respectively. The N—O—N bond has the largest binding energy about −6 eV, which is much larger than that of N—O—Ga (−3.5 eV). Therefore, we can conclude that N atoms would bond with N—O bonds and initiate the growth of GaN.

**Figure 3:**
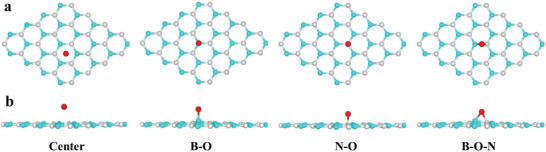
a) Top and b) side view of the four optimized structures of single O atom chemisorbed on an h‐BN monolayer: center (above the center of the h‐BN honeycomb lattice), B—O (only bonding with B), N—O (only bonding with N), and B—O—N (bonding with B and N in form of a bridge). Color coding: Cyan for B, gray for N, and red for O.

**Table 1:** Calculated binding energy (*E*_B_) per O atom adsorbed on h‐BN surface. Here, *E*_B_ is calculated by the following formula: *E*_B_ = *E*_BN‐O_ − *E*_BN_ − *E*_O_, where *E*_BN‐O_, *E*_BN_ = −439.350 eV, and *E*_O_ = −1.606 eV are the energies of monolayer h‐BN adsorbed by one O atom, isolated monolayer h‐BN, and isolated O atom (i.e., a single O atom in the 5 × 5 × 1 supercell), respectively. Negative sign means exothermic

	Center	B—O	N—O	B—O—N
*E*_BN‐O_ [eV]	−441.272	−442.098	−442.522	−443.057
*E*_BN_ [eV]	−439.350	−439.350	−439.350	−439.350
*E*_O_ [eV]	−1.606	−1.606	−1.606	−1.606
*E*_B_ [eV]	−0.316	−1.142	−1.566	−2.101

**Table 2:** Calculated *E*_B_ of various cases of Ga or N atom absorption on h‐BN, where the N—O sites are considered as effective dangling bonds. The *E*_B_ is estimated by *E*_B_ = *E*_Final_ − *E*_Initial_ − *E*_Ga/N_ + *E*_Replaced_, where *E*_Final_, *E*_Initial_, *E*_Ga/N_, and *E*_Replaced_ are the energies of Ga or N atom absorption on h‐BN, h‐BN with N—O bond, single Ga or N atom, and the replaced O atom (for the N—Ga and N—N absorption without O). Negative sign means exothermic

	N—O—Ga	N—O—N	N—Ga	N—N
*E*_Final_ [eV]	−446.253	−451.654	−439.75	−442.793
*E*_Initial_ [eV]	−442.522	−442.522	−442.522	−442.522
*E*_Ga/N_ [eV]	−0.263	−3.124	−0.263	−3.124
*E*_Replaced_ [eV]	0	0	−1.606	−1.606
*E*_B_ [eV]	−3.468	−6.008	1.429	1.247

Now, we are able to illustrate the nucleation of GaN on h‐BN as shown in **Figure** **4**. During HCl treatment, N—OH and B—O—N bonds are generated through the help of hydroxide ions, in which H atoms were then desorbed during the temperature ramping of substrate in the metal–organic chemical vapor deposition (MOCVD) growth. When the Ga and N atoms are supplied, N adatoms prefer to bind with N—O bonds and form the N—O—N bonds. And then, three Ga adatoms bind with each N—O—N bond and form N—O—N—Ga (3) bond and thus initiate the GaN epitaxy. This kind of atomic bond configuration at the interface leads to the epitaxy of Ga‐polarity GaN, which is experimentally confirmed after the growth by chemical etching effect (see Figure S4, Supporting Information). This also indicates that the proposed atomic configurations at the interface are reasonable.

**Figure 4:**
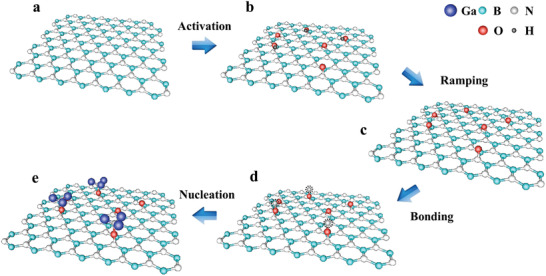
Schematic diagrams of activation of h‐BN and nucleation mechanism of GaN. a) Surface of h‐BN. b) Formation of B—O—N and N—O bonds on h‐BN surface by trapping OH— in HCl solution. c) Desorption of H atoms from activated h‐BN surface (i.e., from N—O—H bonds) during the temperature ramping of substrate. d) Generation of N—O—N bonds on N—O bonds by trapping N atoms. e) Formation of N—O—N—Ga (3) structures by binding three Ga atoms on every N—O—N, indicating the start of Ga‐polarity GaN epitaxy.

The improvement of GaN nucleation on h‐BN not only enhances the surface smoothness of GaN epilayers but also improves their crystalline quality, as shown in **Figure** **5**a,b. The FWHMs of X‐ray diffraction (XRD) *ω*‐rocking curve for the symmetric (0002) and asymmetric (10$\bar{1}$2) planes of 3‐µm‐thick GaN epilayer decrease from 591 and 841 arcsec to 316 and 543 arcsec, respectively. In fact, the GaN film grown on the activated 3‐nm‐thick h‐BN interlayer exhibits similar crystal quality as those grown on Al_2_O_3_ at the same growth condition (see Figure S5a, Supporting Information). But there is an advantage when using h‐BN interlayer, which is strain relaxation in the GaN layer. Figure 5c,d shows the Raman scattering spectra of GaN growth with and without activated h‐BN interlayer. It is shown that strain‐sensitive E_2_ (high) and A_1_ (LO) peaks shift from 570.5 to 569.1 cm^−1^ and from 737.5 to 735.8 cm^−1^, respectively, indicating the relaxation of residual compressive strain in the GaN epilayer.^[^ ^8^, ^47^ ^]^ In fact, the residual compressive strain in the GaN layer can be further relaxed by increasing the thickness of h‐BN interlayer (see Figure S5b, Supporting Information). However, the crystalline quality is also degraded with increasing the h‐BN thickness unfortunately. Anyway, this strain relaxation in the GaN epilayer is definitely helpful to improve the emission of following InGaN quantum wells toward longer wavelength.

**Figure 5:**
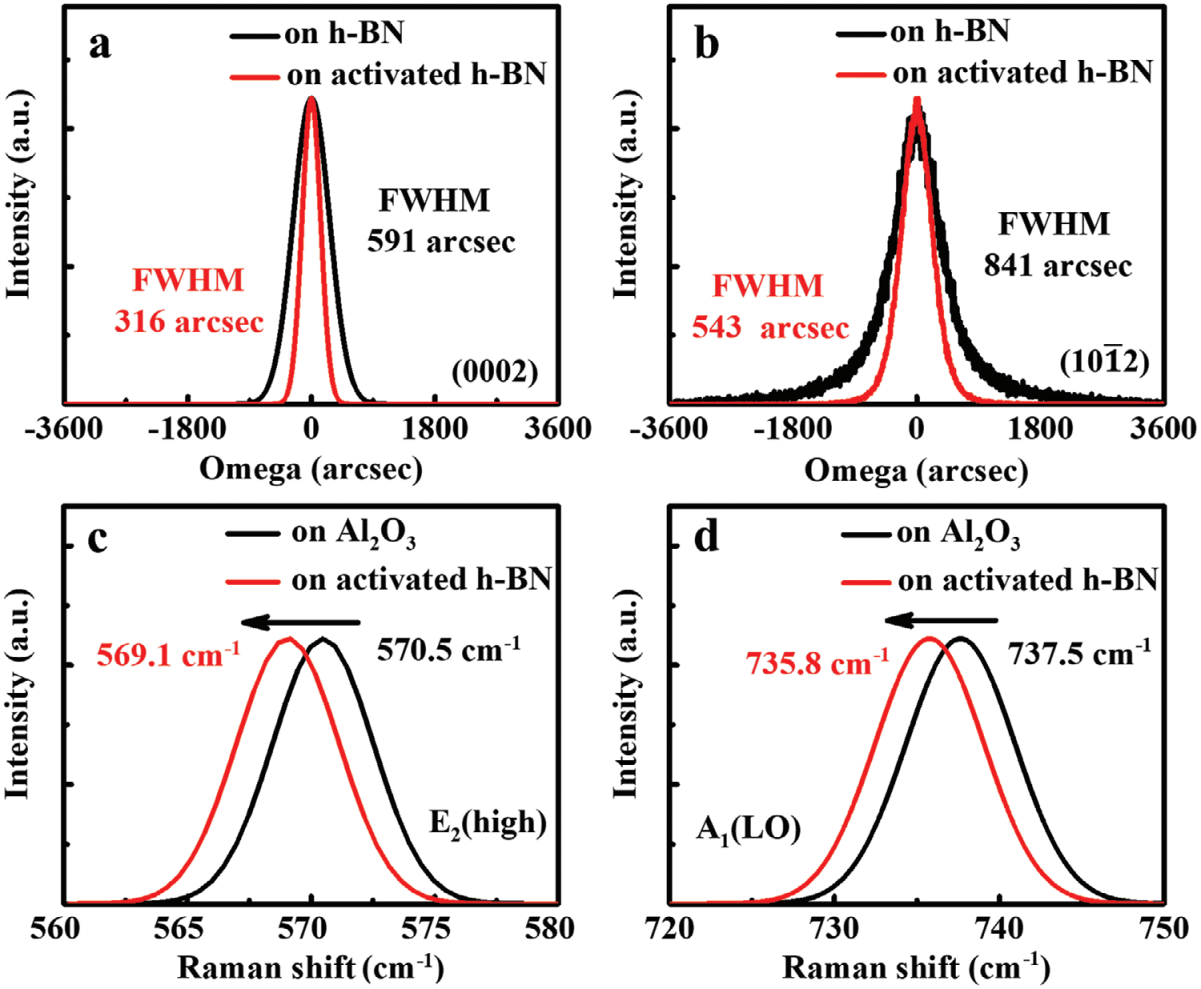
FWHMs of a) (0002)‐ and b) (10$\bar{1}$2)‐plane XRD *ω*‐rocking curves for 3‐µm‐thick GaN films grown on untreated h‐BN/Al_2_O_3_ and activated h‐BN/Al_2_O_3_ substrates. Raman shifts of the c) E_2_ (high) and d) A_1_ (LO) modes of GaN in 3‐µm‐thick GaN films grown on Al_2_O_3_ and activated h‐BN/Al_2_O_3_ substrates. The thickness of h‐BN interlayer is about 3 nm.

Finally, LEDs based on InGaN/GaN MQWs were grown on as‐fabricated undoped GaN/h‐BN/Al_2_O_3_ templates, where the thickness of activated h‐BN and undoped GaN were 3 nm and 3 µm, respectively. For comparison, the LEDs on Al_2_O_3_ were grown in one run. **Figure** **6**a,b exhibits XRD 2*θ*‐*ω* scans for those LED structures, where intense diffraction peaks from GaN epilayer and satellite peaks from the InGaN/GaN MQWs up to the fourth order are observed in both cases, indicating excellent quality and sharp interfaces of the InGaN/GaN MQWs. It is noted that the fitted In composition in the InGaN quantum wells increases from 25% to 28% after using the activated h‐BN interlayer. The AlGaN diffraction peaks come from the electron blocking layer (EBL) in the LED structure. To evaluate the relationship of In composition and strain distribution in InGaN/GaN MQWs, the X‐ray reciprocal space mapping (RSM) scans of the (10$\bar{1}$5) asymmetric reflection are measured, as depicted in Figure 6c,d. The satellite peaks from MQWs vertically line up with the GaN diffraction maximum along *Q*_x_, demonstrating that the InGaN/GaN MQWs are coherently grown on the bottom n‐type GaN layer. As shown by the peak position relative to *Q*_x_, the *n*‐GaN layer grown on the h‐BN template has a larger in‐plane lattice constant than that grown on Al_2_O_3_, indicating a smaller in‐plane compressive strain in the *n*‐GaN layer and InGaN/GaN MQWs region. This result indicates that the biaxial compressive strain within the basal plane in GaN is partially released by using h‐BN interlayer, which could lead to high incorporation efficiency of indium during the growth of InGaN/GaN MQWs. Figure 6e,f demonstrates electroluminescence (EL) spectra for the LEDs with and without using the activated h‐BN interlayer. The LED grown on the 3‐nm‐thick h‐BN interlayer shows a pure green emission with a center wavelength at ∼ 556 nm at typical injection current density of 40 mA·mm^−2^, which is ∼ 26 nm redshift in comparison with that on Al_2_O_3_. It is known that the relaxed compressive strain suppresses the QCSE in InGaN/GaN MQWs, leading to the slight blueshift of emission wavelength and stronger electron–hole wave function overlaps and improved radiative recombination rate.^[^ ^48^, ^49^ ^]^ According to the estimation of the degree of compressive stress relaxation of green LED, the redshift for ∼ 1.1 cm^−1^ of the E_2_ (high) peak belonging to GaN film below MQWs region will lead to the blueshift of emission wavelength of about 2 nm.^[^ ^50^ ^]^ However, the blueshift is compensated since strain relaxation is beneficial to the In incorporation in the InGaN wells and it leads to an emission peak redshift of ∼26 nm. The slight broadening of the linewidth is likely caused by In alloy fluctuations in In‐richer InGaN wells. By the way, a slight blueshift of emission and spectrum broadening are observed in both LEDs with increasing the injection current density, which is most likely induced by the combined effect of the free‐carrier screening of piezoelectric fields and the band filling effect. We also investigated temperature‐dependent photoluminescence (PL) spectra of both LED structures (see Figure S6, Supporting Information). It shows that the insertion of the h‐BN interlayer not only keeps the emission intensity but also tunes the emission toward a longer wavelength, as what we observed in the EL spectra. Notably, the internal quantum efficiency (IQE) of green LED structure on activated h‐BN/Al_2_O_3_ is ∼23% (∼556 nm) that is close to ∼25% (∼530 nm) for the one on Al_2_O_3_. Normally, with the increase of emission wavelength, InGaN‐based LEDs suffer from a systematic drop in efficiency, known as the green gap in III‐nitrides.^[^ ^51^, ^52^ ^]^ Therefore, it is reasonable to believe that the application of h‐BN interlayer provides a promising approach to partially break through the green gap in III‐nitrides, i.e., the epitaxial bottleneck of high In content nitrides, by commercial MOCVD technique.

**Figure 6:**
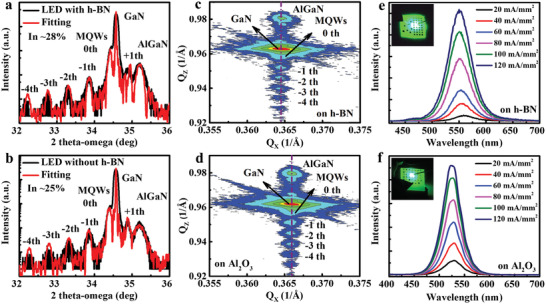
XRD 2*θ*‐*ω* scans of symmetric (0002) plane for InGaN‐based LEDs grown on a) activated h‐BN/Al_2_O_3_ and b) Al_2_O_3_, respectively. RSM scans of the asymmetric (10$\bar{1}$5) reflection for InGaN‐based LEDs grown on c) activated h‐BN/Al_2_O_3_ and d) Al_2_O_3_, respectively. The dashed lines show that InGaN/GaN MQWs coherently grow on *n*‐GaN layer. EL spectra of these InGaN‐based LEDs grown on e) activated h‐BN/Al_2_O_3_ and f) Al_2_O_3_ at different current densities. The insets show the EL images of these LEDs at the injection current density of 40 mA·mm^−2^.

Epitaxy of single‐crystalline GaN films is performed on h‐BN/Al_2_O_3_ substrates. The chemical activation of h‐BN provides B—O—N and N—O bonds, where the latter ones act as effective artificial dangling bonds for GaN nucleation, resulting in successful epitaxy of Ga‐polarity GaN. The h‐BN interlayer modifies the residual compressive strain in GaN film and thus improves indium incorporation during the following growth of InGaN quantum wells, leading to the achievement of pure green light‐emitting diodes. This work provides an effective way for III‐nitrides epitaxy on h‐BN and a possible route to overcome the epitaxy bottleneck of high indium composition InGaN‐based light‐emitting devices.

## Experimental Section

##### Fabrication of Crystalline h‐BN

The 2‐in. (0001) Al_2_O_3_ substrates were transferred into a plasma‐assisted molecular beam epitaxy (PA‐MBE) chamber and then thermally cleaned for 30 min at 900 °C. Subsequently, the BN was deposited on the entire surface of the Al_2_O_3_ at 900 °C with a growth rate of 0.2 nm·min^−1^. Through high temperature annealing at 1700 °C in N_2_ ambient for 2 h, the quality of that BN are much improved (see Figure S1, Supporting Information). That crystalline h‐BN was then used as a template for GaN epitaxy.

##### Chemical Activation of Crystalline h‐BN

The h‐BN/Al_2_O_3_ substrates were put in acetone, ethanol, and deionized water solution (30 mL) for 3 min to clean successively. Then, these h‐BN/Al_2_O_3_ substrates were dipped into hydrochloric acid solution for 10 min for activation treatment. Finally, the treated samples were rinsed by deionized water and dried with flowing nitrogen.

##### Epitaxial Growth of GaN Films and InGaN‐Based LEDs

GaN films were grown on the activated h‐BN/Al_2_O_3_ templates by an AIXTRON 3 × 2 in. FT MOCVD system. H_2_ was used as the carrier gas. Prior to the deposition, the substrates were thermally cleaned in H_2_ ambient at 1100 °C for 5 min. The growth began with a low temperature GaN (LT‐GaN) nucleation layer deposited at 530 °C. Then, the growth temperature ramped up to 1050 °C for the growth of the high temperature GaN (HT‐GaN) layer. The InGaN‐based multiple quantum wells (MQWs) LED structures were then grown on GaN/h‐BN/Al_2_O_3_. The thickness of h‐BN and GaN were 3 nm and 3 µm, respectively. The LED structure consisted of a Si‐doped GaN layer (1.5 µm), five periods of InGaN/GaN MQWs, a Mg‐doped AlGaN EBL (20 nm), a Mg‐doped GaN layer (200 nm), and a heavy Mg‐doped cap layer (10 nm). The Si‐doped GaN layer was grown at 1050 °C with an electron concentration of about 3 × 10^18^ cm^−3^. Subsequently, InGaN/GaN MQWs were grown at 712 °C/840 °C with 2.5 nm InGaN quantum well (QW) layers and 15 nm GaN quantum barrier (QB) layers. Both QW and QB layers were unintentionally doped. The Mg‐doped Al_0.3_Ga_0.7_N EBL ([Mg] ≈ 3.7 × 10^20^ cm^−3^) was deposited at 950 °C. At last, the Mg‐doped GaN ([Mg] ≈ 5.0 × 10^20^ cm^−3^) and heavy Mg‐doped cap layer ([Mg] ≈ 1.0 × 10^21^ cm^−3^) were grown at 940 °C. For comparison, the same LED structure was also grown on Al_2_O_3_. The LEDs on Al_2_O_3_ and activated h‐BN/Al_2_O_3_ were grown in one run.

##### Characterization

XRD measurement was performed by X'Pert3 MRD system using Cu K*α*_1_ X‐ray source. Surface morphology was measured by atomic force microscopy (AFM) in tapping mode (Bruker Dimension ICON‐PT) and scanning electron microscopy (SEM). XPS was performed to quantitatively estimate the chemical states of h‐BN before and after HCl treatment. The XPS spectra were measured by an ESCALab 250 Analytical XPS spectrometer with a monochromatic X‐ray source (Al K*α*, *hν* = 1486.6 eV). The binding energies of the spectra were referred to that of the C 1s peak at ∼284.8 eV. Strain in GaN was characterized by Raman scattering spectroscopy. The EL spectrum of the GaN‐based LEDs was obtained at a driving current of 70 mA by using the home‐made acquisition equipment at room temperature including a lock‐in amplifier system and photomultiplier tube. The PL spectra of these LED structures were measured by a PL system which includes a He‐Cd laser (325 nm, 30 mW) as an excitation source, a Jobin Yvon iHR550 spectrometer, a Syncerity charge coupled device (CCD), and a closed circle helium cryostat.

##### Simulation

DFT calculations were implemented by the Vienna ab initio Simulation Package (VASP) code. The projector augmented wave (PAW) pseudopotentials were used for electron–ion interaction. The generalized gradient approximation (GGA) was used to the exchange‐correlation functional as proposed by Perdew–Burke–Ernzerhof (PBE). The energy cutoff for plane wave expansion is 520 eV. In the calculation, (5 × 5 × 1) Monkhorst–Pack k‐points were used and spin polarization was considered. This monolayer h‐BN unit cell model contained a 20 Å vacuum layer along the *c*‐direction. The lattice parameters of the h‐BN unit cell were *a* = *b* = 2.513 Å, *α* = *β* = 90°, *γ* = 120°. More detailed information was provided in calculation part (i.e., Binding energy computation) of the Supporting Information.

##### Statistical Analysis

The data were obtained by Nano Measurer 1.2 for Windows. In order to ensure the repeatability of the nucleation experiment, different regions were taken to calculate the nucleation density, and sample size (*n*) for each statistical analysis was 3. Among the GaN grain density on activated h‐BN/Al_2_O_3_ is about 69 ± 1.6 µm^−2^, and the density on untreated h‐BN/Al_2_O_3_ is about 24 ± 2.1 µm^−2^.

## Conflict of Interest

The authors declare no conflict of interest.

## Supporting information

Supporting Information
