# Supplementary material for: Hexagonal BN‐Assisted Epitaxy of Strain Released GaN Films for True Green Light‐Emitting Diodes
Source: Adv Sci (Weinh). 2020 Sep 27;7(21):2000917. doi: 10.1002/advs.202000917 (PMC7610270; doi:10.1002/advs.202000917)
Supplement: Supplementary file 1 — Supporting Information [file ADVS-7-2000917-s001.pdf]

© 2020 Wiley-VCH GmbH

ADVANCED SCIENCE
Open Access

Supporting Information

for *Adv. Sci.*, DOI: 10.1002/advs.202000917

Hexagonal BN-Assisted Epitaxy of Strain Released GaN Films for True Green Light-Emitting Diodes

Fang Liu, Ye Yu, Yuantao Zhang, Xin Rong, Tao Wang, Xiantong Zheng, Bowen Sheng, Liuyun Yang, Jiaqi Wei, Xuepeng Wang, Xianbin Li, Xuelin Yang, Fujun Xu, Zhixin Qin, Zhaohui Zhang, Bo Shen and Xinqiang Wang**

Supporting Information

Hexagonal BN-Assisted Epitaxy of Strain Released GaN Films for True Green Light-Emitting Diodes

Fang Liu, Ye Yu, Yuantao Zhang, Xin Rong, Tao Wang, Xiantong Zheng, Bowen Sheng, Liuyun Yang, Jiaqi Wei, Xuepeng Wang, Xianbin Li, Xuelin Yang, Fujun Xu, Zhixin Qin, Zhaohui Zhang, Bo Shen and Xinqiang Wang**

(E-mail: zhangyt@jlu.edu.cn; wangshi@pku.edu.cn)

S1. Achievement of wafer-scale crystalline h-BN on Al_2O_3

As depicted in **Figure S1**, 3-nm-thick BN grown on Al_2O_3 using plasma-assisted molecular beam epitaxy (PA-MBE) is disordered due to the low growth temperature below 1000 °C, as shown by the typical hollow reflection high-energy electron diffraction (RHEED) patterns. Thermodynamically stable crystalline h-BN could be obtained at wafer scale (2 inch) by high temperature annealing as previously reported.[1] The crystalline h-BN is confirmed by streaky RHEED patterns.

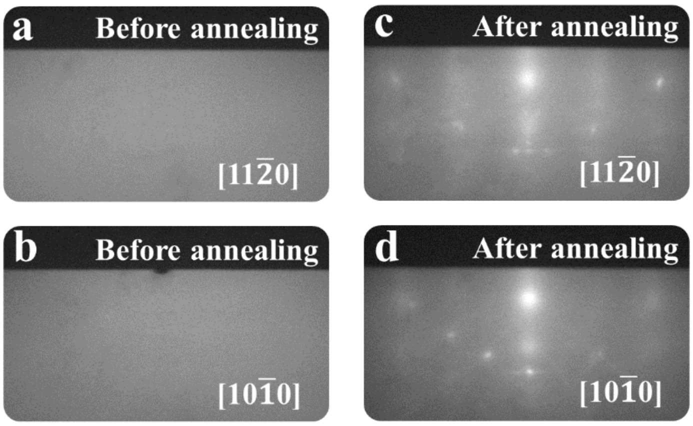

Fig. S1. a-d) RHEED patterns of 3-nm-thick BN films before and after high temperature annealing at 1700 °C for 2h in N_2 ambient.

S2. Statistics of the density and size of the GaN grains on h-BN

The 30-nm-thick low temperature GaN (LT-GaN) was deposited on untreated h-BN/Al_2O_3 and activated h-BN/Al_2O_3 substrates, respectively. With a growth interruption after LT-GaN annealing, the density and size of GaN grains on these substrates are calculated, as shown in **Figure S2**. The overall GaN grain density increases by about threefold from 24 μm^{-2} to 69 μm^{-2} after surface activation. The density of GaN grains with smaller size (≤ 100 nm) is almost unchanged, indicating that this part of GaN domains is not affected by HCl treatment of h-BN. In contrast, the density of GaN grains with larger size (>100 nm) is obviously increased from 4 μm^{-2} to 48 μm^{-2}. It reveals that HCl treatment of h-BN is mainly beneficial to the nucleation growth of GaN grains with larger size.

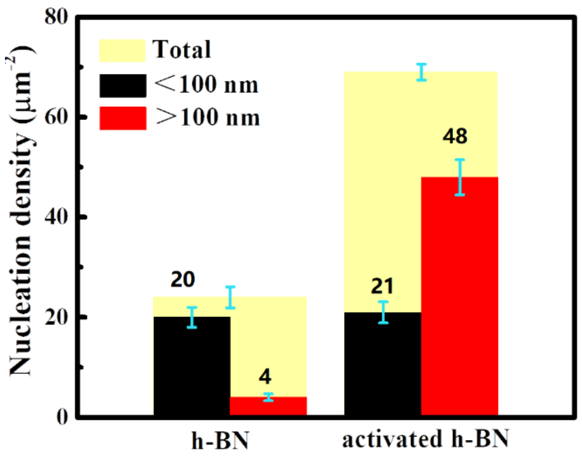

Fig. S2. Statistics of the density and size of the GaN grains on untreated h-BN/Al_2O_3 and activated h-BN/Al_2O_3, respectively. The thickness of h-BN is ~3 nm.

S3. Chemical-activated process of h-BN

As shown in **Figure S3**a, the intensity of B 1s and N 1s signals decreases significantly after HCl treatment. Since XPS technology is very sensitive to the element distribution on the sample surface, it is reasonable to believe that part of the surface of h-BN is damaged, leading B and N atoms to desorb from the surface and leaving some unsaturated dangling bonds.[2] Notably, this process can occur on entire h-BN surface. By the way, the stable chemical state of O 1s, coming from the Al_2O_3 substrates, is also confirmed in Figure S3b and c after the HCl treatment. This is because X-ray will pass through the h-BN which owes limited thickness (~3 nm), and interact with Al_2O_3 substrate.

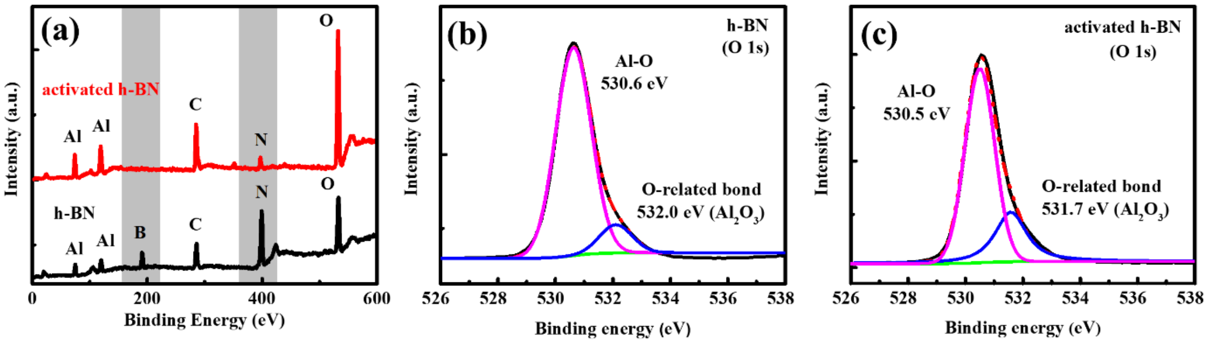

Fig. S3. a) XPS survey spectra of h-BN/Al_2O_3 substrate before and after HCl treatment. These XPS survey spectra of h-BN/Al_2O_3 both include a contribution from peaks of Al 2p (~74 eV), Al 2s (~120 eV), B 1s (~190 eV), C 1s (~285 eV), N 1s (~398 eV) and O 1s (~531 eV). The stable O 1s peak existing in b) untreated h-BN and c) activated h-BN consists of Al-O bonds (~530.5 eV) and O-related bonds (~531.7 eV), which both come from Al_2O_3 substrate. The thickness of h-BN is about 3 nm.

S4. Polarity determination of GaN by chemical etching

The lattice polarity of GaN films grown on activated h-BN/Al_2O_3 was confirmed by wet chemical etching. **Figure S4** shows surface morphologies of GaN film before and after chemical etching by KOH solution. The GaN surface keeps flat, which coincides with the

etching behavior of Ga-polarity GaN as previously reported. It confirms that the GaN is Ga-polarity.[3,4]

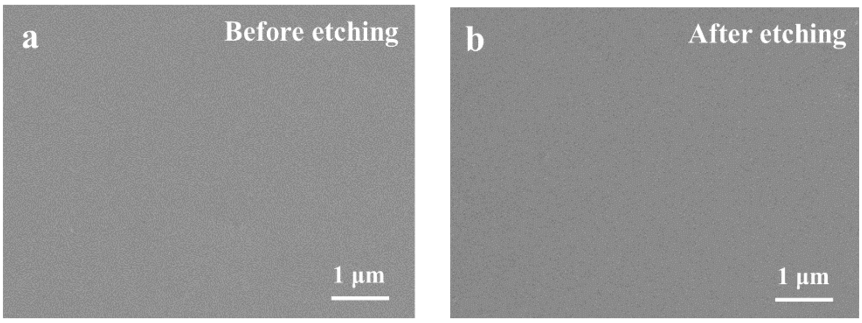

Fig. S4. SEM images of 3-μm-thick GaN epilayers grown on activated h-BN/Al_2O_3 a) before and b) after KOH etching. The thickness of h-BN is ~3 nm.

S5. Effect of h-BN thickness on the stress distribution and crystal quality of GaN films

As shown in **Figure S5**, the structural quality and strain distribution of GaN layers grown on h-BN had been investigated. Figure S5a shows the FWHM of rocking curves of (0002) symmetric and $(10\bar{1}2)$ asymmetric planes as a function of annealed h-BN interlayer thickness. It is shown that the GaN layer grown on 3-nm-thick annealed h-BN interlayer shows comparable crystal quality as that grown on Al_2O_3. It is also observed that the structural quality degrades with increasing thickness of the annealed h-BN up to 10 nm, indicated by the larger FWHMs in both (0002) and $(10\bar{1}2)$ rocking curves of GaN layers. This may be related to two factors. On one hand, it can be explained by the strain relaxation since compressive strain is helpful to annihilate threading dislocations.[5,6] On the other hand, with the increase of h-BN thickness, the effect of the substrate on the regular arrangement of the upper GaN is further shielded, resulting in the unsatisfactory GaN crystal lattice structure.[7] As shown in Figure S5b, the strain-sensitive E_2(high) and A_1(LO) peaks shift from ~570.5 to 568.2 cm^{-1} and from 737.5 to 734.4 cm^{-1} respectively, as the thickness of h-BN interlayer

increases to 10 nm. This demonstrates that compressive strain in GaN is continually relaxed with increasing thickness of h-BN interlayer.

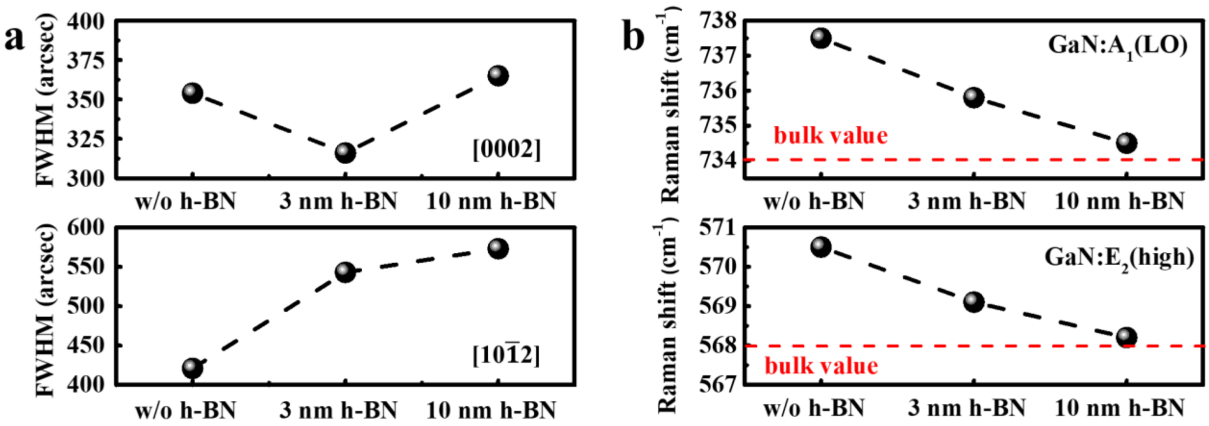

Fig. S5. a) FWHMs of (0002)- and (10$\bar{1}$2)-plane XRCs for GaN films grown on activated h-BN with different thicknesses. b) Raman frequency shifts of A_1(LO) and E_2(high) modes of GaN films grown on activated h-BN with different thicknesses. The peak positions of A_1(LO) and E_2(high) modes coming from bulk GaN are 734 cm^{-1} and 568 cm^{-1}, which are labeled by the red lines in b) and used as reference.

S6. Temperature-dependent photoluminescence (PL) spectra of the InGaN-based LEDs

As shown in **Figure S6**a, the green LED structure consists of an un-doped GaN layer, Si-doped GaN layer, 5-period InGaN/GaN multiple-quantum-wells (MQWs), a p-type AlGaN electron blocking layer (EBL) and Mg-doped p-GaN/p^+-GaN hole-injection layers. As depicted in Figure S6b and c, the spectra show that PL intensity of MQWs gradually increases with decreasing temperature and reaches a maximum at 10 K. It is shown that the peak position of the MQWs emission is ~550.7 nm for the structure with activated h-BN interlayer at 10 K, while that is ~523.1 nm for the structure without h-BN. Besides, the peak intensity of the LED grown on activated h-BN is slightly stronger than the LED grown on Al_2O_3 at the same excitation power. The FWHM of the emission peak increases from 39.7 nm to 55.6 nm

with the introduction of h-BN interlayer, which is likely related to the higher In-content incorporation in MQWs. Notably, internal quantum efficiency (IQE) of green LED structure with activated h-BN interlayer is ~23% that is close to ~25% for the one without h-BN. Normally, it is very difficult to grow green LEDs with longer emission wavelength, which is named green gap in III-nitrides.[8-10] In other word, the IQE of green LEDs rapidly decreases as the increase of In content, i.e., the longer emission wavelength. Therefore, the ~23% of IQE for the green LEDs on h-BN definitely shows the achievement of excellent pure green light emitting diodes, and provides a potential route to achieve high In content LEDs.

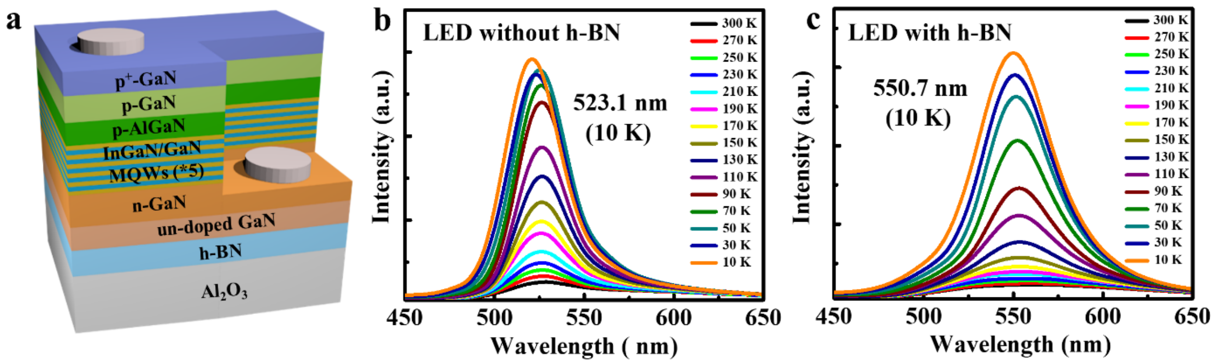

Fig. S6. a) Schematic illustration of the green InGaN-based LED structure grown on activated h-BN/Al_2O_3 substrate. Temperature-dependent PL spectra of InGaN-based green LEDs on the Al_2O_3 substrates b) without and c) with the activated h-BN interlayer. The thickness of h-BN is about 3 nm.

S7. Binding energy computation

The density functional theory (DFT) calculations are implemented by the Vienna ab initio Simulations Package (VASP) code. The projector augmented wave (PAW) pseudopotentials are used for electron-ion interaction. We use the generalized gradient approximation (GGA) to the exchange-correlation functional as proposed by Perdew-Burke-Ernzerhof (PBE). The energy cutoff for plane wave expansion is 520 eV. In the calculations,

(5×5×1) Monkhorst-Pack k-points are used and spin polarization has been considered. Our monolayer h-BN unit cell model contains a 20 Å vacuum layer along c direction. The lattice parameters of the BN unit cell are a = b = 2.513 Å, α = β = 90°, γ = 120°. For the O atoms adsorption model, we use a 5×5×1 h-BN supercell which contains 25 B atoms, 25 N atoms, and 1 O atom. The binding energy (E_B) of O atom on monolayer h-BN is calculated by $E_B = E_{BN\text{-}O} – E_{BN} – E_O$, where $E_{BN\text{-}O}$, E_{BN} = -439.350 eV, and E_O = -1.606 eV are the energies of monolayer h-BN adsorbed by O, isolated monolayer h-BN, and isolated O atom (i.e. a single O atom in the 5×5×1 supercell), respectively, with the same lattice parameters. The total energy and binding energy of O atoms adsorbed monolayer h-BN structure are summarized in **Table 1**.

Various cases of Ga or N atom absorptions on monolayer h-BN to trigger GaN nucleation and growth are further analyzed by DFT total energy calculations. Based on the formation of N-O bond as the nucleation sites on h-BN, the calculation of N-O-Ga, N-O-N, N-Ga, and N-N binding energy is considered. The binding energy (E_B) is estimated by $E_B = E_{Final} – E_{Initial} – E_{Ga/N} + E_{Replaced}$, where E_{Final}, $E_{Initial}$, $E_{Ga/N}$, $E_{Replaced}$ are the energies of Ga/N absorption on h-BN, h-BN with N-O bonding, single Ga or N atom, and the replaced O atom (for the N-Ga and N-N absorption without O). As shown in **Table 2**, the calculation results show N-O-N bond is the most stable on the h-BN surface, hence triggers the nucleation of the nitrides and finally gets the Ga-polar films. All these calculations have included spin polarization.

Reference:

[1] F. Liu, X. Rong, Y. Yu, T. Wang, B. W. Sheng, J. Q. Wei, S. F. Liu, J. J. Yang, F. Bertram, F. J. Xu, X. L. Yang, Z. H. Zhang, Z. X. Qin, Y. T. Zhang, B. Shen, X. Wang, *Appl Phys Lett.* **2020**, 116, 142104 (2020).

[2] Q. Wu, J. Yan, L. Zhang, X. Chen, T. Wei, Y. Li, Z. Liu, X. Wei, Y. Zhang, J. Wang, J. Li, *CrystEngComm.* **2017**, *19*, 5849.

[3] S. Jung, K. R. Song, S. N. Lee, H. Kim, *Adv. Mater.* **2013**, *25*, 4470.

[4] D. Li, M. Sumiy, S. Fuke, D. Yang, D. Que, Y. Suzuki, Y. Fukuda, *J. Appl. Phys.* **2001**, *90*, 4219.

[5] D. M. Follstaedt, S. R. Lee, A. A. Allerman, J. A. Floro, *J. Appl. Phys.* **2009**, *105*, 083507

[6] J. Cheng, X. Yang, L. Sang, L. Guo, A. Hu, F. Xu, N. Tang, X. Wang, B. Shen, *Appl. Phys. Lett.* **2015**, *106*, 142106.

[7] J. Yu, L. Wang, Z. Hao, Y. Luo, C. Sun, J. Wang, Y. Han, B. Xiong, H. Li, *Adv. Mater.* **2020**, 32, 1903407.

[8] M. A. Maur, A. Pecchia, G. Penazzi, W. Rodrigues, A. D. Carlo, Phys. Rev. Lett. 2016, 116, 027401.

[9] S. Saito, R. Hashimoto, J. Hwang, S. Nunoue, *Appl. Phys. Express* **2013**, *6*, 111004.

[10] Y. Jiang, Y. Li, Y. Li, Z. Deng, T. Lu, Z. Ma, P. Zuo, L. Dai, L. Wang, H. Jia, W. Wang, J. Zhou, W. Liu, H. Chen, *Sci. Rep.* **2015**, *5*, 10883.
